# 

**Published:** 1835-07-01

**Authors:** 


### CONTENTS

OF THE

### MEDICO-CHIRURGICAL REVIEW,

No. XLV. JULY 1, 1835.

[BEING No. V. OF A DECENNIAL SERIES.]

### REVIEWS.

PHTHISIS.

I.

1. Pathological Researches on Phthisis. By M. LOUIS .. .. .. .. .. .. ..
2. A Treatise on Tubercular Phthisis. By JAMES CLARK, M.D. .. .. .. .. ..
3. Consumption, why so fatal? By JOHN TYRRELL, Esq. .. .. .. .. .. .. } 1 to 45

II.

Transactions of the Provincial Medical and Surgical Association. Vol. III. 1835
Mr. James on the Causes of good or bad Stumps .. .. .. .. .. .. .. } 45

III.

Th[illegible] Epidemics of the Middle Ages. By J. HECKER,—translated by Dr. BABINGTON .. 49

IV.

Sketch of the History of Medicine, from its Origin to the 19th Century. By J. BOS[illegible]CK, M.D. .. .. .. .. .. .. .. .. .. .. .. .. .. .. .. .. 53

V.

Transactions of the Medical Society of New York. Vol. 1835.
1. On Delirium Tremens. By Dr. CROSS .. .. .. .. .. .. .. .. .. .. 57
2. Puerperal Fever. By Dr. EIGHTS.. .. .. .. .. .. .. .. .. .. .. .. .. 59
3. Dr. SPENCER on Cholera .. .. .. .. .. .. .. .. .. .. .. .. .. .. 60

VI.

M. VELPEAU on Puerperal Convulsions .. .. .. .. .. .. .. .. .. .. .. .. 61
1. Eclampsia Hysterica, (65). 2. The Tetanic Eclampsia, (65). 3. The Epileptic, (65). 4. The Apoplectic form, (66-7.)

VII.

DISEASES OF THE HEART AND CHEST.

1. The Pathology and Diseases of the Chest, &c. By Dr. WILLIAMS .. . . ..

2. On Diseases of the Heart. By Dr. HOPE .. .. .. .. .. .. .. .. .. .. } 69 to 89

VIII.

Clinique Medicale. By Dr. ANDRAL. (Diseases of the Encephalon.) .. 89 to 106

IX.

A Treatise on Insanity, and other Disorders of the Mind. By Dr. PRICHARD .. .. 106

### X.

### Works on Pathological Anatomy.

1. Dr. Carswell's Illustrations of the Elementary Forms of Disease .. .. .. }

2. Anatomie Pathologique du Corps Humain. Par J. Cruveilhier. (Maladies du Fœtus) .. .. .. .. .. .. .. .. .. .. .. .. .. .. .. } 122 to 144

### PERISCOPE.

### PART I.

### Spirit of the English Periodicals, and Notices of English Medical Literature.

1. Catalepsy—remarkable case of .. .. .. .. .. .. .. .. .. .. .. .. .. .. 145
2. Galvanic Experiments. By Dr. Dunbar .. .. .. .. .. .. .. .. .. .. 146
3. Dr. Christian on Spitting of Blood .. .. .. .. .. .. .. .. .. .. .. 150
4. Instructive Case of Monomania .. .. .. .. .. .. .. .. .. .. .. .. 150
5. Dr. Elliotson on Kreosote .. .. .. .. .. .. .. .. .. .. .. .. .. 151
6. Unique Disease of the Heart .. .. .. .. .. .. .. .. .. .. .. .. 152
7. Mr. Dendy's Oration before the London Medical Society .. .. .. .. .. .. .. 153
8. Obscure Pericarditis—Dilatation of the Heart—Œdema of the Fauces, &c. By Dr. S. Jackson .. .. .. .. .. .. .. .. .. .. .. .. .. .. 156
9. The Sphygmometer. By Dr. Julius Herisson .. .. .. .. .. .. .. .. .. 159
10. Coroner's Inquest—disgraceful conduct .. .. .. .. .. .. .. .. .. .. 160
11. Curious Case of Paraplegia .. .. .. .. .. .. .. .. .. .. .. .. 160
12. Medical Tuition—Circular of the Bartholomew School .. .. .. .. .. .. .. 162
13. The Mirage. By Lieutenant Burnes .. .. .. .. .. .. .. .. .. .. .. 163
14. Intussusception, with Stercoraceous Vomiting, treated by Inflation .. .. .. 164
15. Prejudices against Fish Diet. By Lieut. Burnes .. .. .. .. .. .. .. .. 165
16. Œsophagitis .. .. .. .. .. .. .. .. .. .. .. .. .. .. .. .. .. 166
17. Enteritis? Strictures on a Case of, reported by Dr. Graves .. .. .. .. .. .. 166
18. Physical Signs *versus* Ordinary Symptoms. Organic Disease of the Heart and Kidneys—Medical Polity. Debates on the subject. By Dr. James Johnson, 168
19. Pathological Anatomy, continued from page 144.
 1. Diseases of the Lungs and Thymus Gland .. .. .. .. .. .. .. .. .. 172
 2. Diseases of the Mouth and Pharynx .. .. .. .. .. .. .. .. }
 3. Follicular Ulceration of the Stomach .. .. .. .. .. .. .. .. } 173
 4. Diseases of the Spinal Marrow .. .. .. .. .. .. .. .. .. }
 5. Hypertrophy, &c. &c. .. .. .. .. .. .. .. .. .. .. .. .. }
20. On the Management of Gonorrhœa. By the Junior Editor .. .. .. .. }
 Critical Review of Mr. Wallace's Doctrines and Opinions.. .. .. .. .. } 177
21. Remarkable Case of Phthisis, Pneumo-thorax, &c. By Sir D. J. H. Dickson, M.D. 187
22. Last Curriculum of the Society of Apothecaries .. .. .. .. .. .. .. .. 189
23. Extensive Disease of various parts, with a fine Illustration of their Pathological Phenomena. By R. Billis, Esq. .. .. .. .. .. .. .. .. .. .. .. } 189
24. Case of apparently Secondary Symptoms after Gonorrhœa. By H. J. Johnson .. 191

### PART II.

### Spirit of the Foreign Periodicals.

25. Abstract of M. Bouillaud's Clinical Course at La Pitié, 1834 .. .. .. .. .. .. 193
 Pleuro-pneumonia .. .. .. .. .. .. .. .. .. .. .. .. .. .. .. 193
 Pleuritis .. .. .. .. .. .. .. .. .. .. .. .. .. .. .. .. .. 195
 Acute Rheumatism .. .. .. .. .. .. .. .. .. .. .. .. .. .. .. 195

Erysipelas .. .. .. .. .. .. .. .. .. .. .. .. .. .. .. 196
Enteritis .. .. .. .. .. .. .. .. .. .. .. .. .. .. .. 197
Peritonitis .. .. .. .. .. .. .. .. .. .. .. .. .. .. .. 199
26. M. Louis on the Effects of Venæsection in Pneumonia .. .. .. .. .. .. .. 200
M. Donné's Letter on the above Subject .. .. .. .. .. .. .. .. .. .. 203
27. Interesting Case of Chronic Pleurisy, with Pneumo-thorax, &c. .. .. .. .. .. 206
Cases and Commentaries .. .. .. .. .. .. .. .. .. .. .. .. 209
28. Case of Vomica, or Abscess of the Lungs, cured .. .. .. .. .. .. .. .. 213
29. On the Treatment of Hooping-Cough by Revulsives .. .. .. .. .. .. .. 215
30. M. Andral on Gastro-Enteritis and Follicular Enteritis .. .. .. .. .. .. .. 217
31. On Methodic Compression, as a Remedial Agent in Dropsies. By M. Bretonneau, 223
32. Treatment of Gonorrhœal Ophthalmia .. .. .. .. .. .. .. .. .. .. .. 228
M. Sanson's Treatment of Purulent Ophthalmia .. .. .. .. .. .. .. 232
33. Acute Rheumatism, treated with large Doses of the Tartrate of Antimony .. .. 235
34. Scene in the French Academy—Homœopathy .. .. .. .. .. .. .. .. .. 239

### PART III.

### Clinical Review and Hospital Reports.

Birmingham Infirmary.

35. Report of Out-Cases during the Year 1834, with Tables, Statistical Observations, &c. By Mr. Parsons and Mr. Ryland.
Mr. Parsons' Report .. .. .. .. .. .. .. .. .. .. .. .. .. .. 241
Mr. Ryland's Report .. .. .. .. .. .. .. .. .. .. .. .. .. . 245

Sir P. Dun's Hospital.

36. Case of Hydrops Pericardii. By Dr. Law .. .. .. .. .. .. .. .. .. .. 248
Effusion into the Pericardium and Pleura .. .. .. .. .. .. .. .. .. .. 249
Pericarditis from Rheumatism .. .. .. .. .. .. .. .. .. .. .. .. 250

Birmingham Eye Infirmary.

37. Annual Report, &c. By R. Middlemore, Esq. .. .. .. .. .. .. .. .. .. 251
1. Development of Cornea .. .. .. .. .. .. .. .. .. .. .. .. .. 251
2. Opacity of Cornea .. .. .. .. .. .. .. .. .. .. .. .. .. .. 252
3. Staphyloma .. .. .. .. .. .. .. .. .. .. .. .. .. .. .. 253
4. Cataract .. .. .. .. .. .. .. .. .. .. .. .. .. .. .. .. 253
5. Amaurosis .. .. .. .. .. .. .. .. .. .. .. .. .. .. .. 254

Glasgow Infirmary.

38. Report on Tumors of the Abdomen. By J. Macfarlane, M.D. .. .. .. .. .. .. 254
1. Tumors confined to the Abdominal Parietes. Cystic Sarcoma .. . .. 256
2. Tumors of the Peritoneum, Omentum, and Mesentery .. .. .. .. .. 261
3. Tumors from Alvine Concretions .. .. .. .. .. .. .. .. .. .. .. 263
4. Ovarian Tumors .. .. .. .. .. .. .. .. .. .. .. .. .. .. 264
39. French Opinions on English Hospital Surgeons and Surgery .. .. .. .. .. .. 266
40. M. Louis' Essay on Clinical Instruction .. .. .. .. .. .. .. .. .. .. 268
41. Dr. Carbutt's Report on Dropsy .. .. .. .. .. .. .. .. .. .. .. .. 272

Lock Hospital.

42. Mr. H. J. Johnson's Cases of Venereal Affections of the Fœtus .. .. .. .. .. 277

St. George's Hospital.

43. New Laws .. .. .. .. .. .. .. .. .. .. .. .. .. .. .. 281

### Miscellanies.

44. Mr. Howard's Case of doubtful Tumor of the Thigh.. .. .. .. .. .. .. .. 282
45. Acalephæ .. .. .. .. .. .. .. .. .. .. . .. .. .. .. .. .. 284
46. On the Number of the Pulsations of the Heart in the Fœtus. By Dr. Fleming .. 284
47. Medical Associations .. .. .. .. .. .. .. .. .. .. .. .. .. .. .. 285
48. Medical Reform .. .. .. .. .. .. .. .. .. .. .. .. .. .. . 286

Bibliographical Record.. .. .. .. .. .. .. .. .. .. .. .. .. .. .. 286

### Extra-Limites.

Curriculum of the Apothecaries' Company .. .. .. .. .. .. .. .. .. .. 289

